# A Targeted Serum Metabolomics GC-MS Approach Identifies Predictive Blood Biomarkers for Retained Placenta in Holstein Dairy Cows

**DOI:** 10.3390/metabo11090633

**Published:** 2021-09-17

**Authors:** Guanshi Zhang, Dawid Tobolski, Grzegorz Zwierzchowski, Rupasri Mandal, David S. Wishart, Burim N. Ametaj

**Affiliations:** 1 Department of Agricultural, Food and Nutritional Science, University of Alberta, Edmonton, AB T6G 2P5, Canada; zhangg3@uthscsa.edu (G.Z.); grzegorz.zwierzchowski@uwm.edu.pl (G.Z.); 2 Center for Renal Precision Medicine, Division of Nephrology, Department of Medicine, The University of Texas Health, San Antonio, TX 78229, USA; 3 Audie L. Murphy Memorial VA Hospital, South Texas Veterans Health Care System, San Antonio, TX 78229, USA; 4 Faculty of Veterinary Medicine, University of Warmia and Mazury, 14 Oczapowskiego str., 10-718 Olsztyn, Poland; dawid.tobolski@uwm.edu.pl; 5 Faculty of Biology and Biotechnology, University of Warmia and Mazury, 1a Oczapowskiego str., 10-719 Olsztyn, Poland; 6 Department of Biological and Computer Sciences, University of Alberta, Edmonton, AB T6G 2P5, Canada; rmandal@ualberta.ca (R.M.); dwishart@ualberta.ca (D.S.W.)

**Keywords:** retained placenta, dairy cow, metabolomics, biomarkers, GC/MS, amino acids

## Abstract

The retained placenta is a common pathology of dairy cows. It is associated with a significant drop in the dry matter intake, milk yield, and increased susceptibility of dairy cows to metritis, mastitis, and displaced abomasum. The objective of this study was to identify metabolic alterations that precede and are associated with the disease occurrence. Blood samples were collected from 100 dairy cows at −8 and −4 weeks prior to parturition and on the day of retained placenta, and only 16 healthy cows and 6 cows affected by retained placenta were selected to measure serum polar metabolites by a targeted gas chromatography–mass spectroscopy (GC-MS) metabolomics approach. A total of 27 metabolites were identified and quantified in the serum. There were 10, 18, and 17 metabolites identified as being significantly altered during the three time periods studied. However, only nine metabolites were identified as being shared among the three time periods including five amino acids (Asp, Glu, Ser, Thr, and Tyr), one sugar (myo-inositol), phosphoric acid, and urea. The identified metabolites can be used as predictive biomarkers for the risk of retained placenta in dairy cows and might help explain the metabolic processes that occur prior to the incidence of the disease and throw light on the pathomechanisms of the disease.

## 1. Introduction

Retained placenta (RP) affects about 1.3–39.2% of dairy cows in a herd [1]. It is defined as retention of fetal membranes 24 h or longer after parturition [2]. Retained placenta is one of the main causes of endometritis in dairy cows and is associated with significant economic losses [3]. Moreover, RP is a direct risk factor for postpartum metabolic and reproductive disorders, which may affect the subsequent reproductive performance of dairy cows [4]. The most common diagnostic indicators of RP are degenerating, discolored, and fetid membranes hanging from the vulva > 24 h postpartum [5]. It should be pointed out that it is always too late and costly to deal with RP when the disease already occurs. Indeed, dairy cattle with RP must be immediately culled. Therefore, the identification of predictive biomarkers for RP is of utmost benefit to dairy producers.

The etiopathology of RP in cows is complex and incompletely understood. A recent study conducted by our group revealed that cows with RP experienced elevated concentrations of several variables related to innate immunity and carbohydrate metabolism at −8 and −4 weeks prior to the occurrence of disease compared to healthy cows [5,6]. Therefore, it would be of great interest to study metabolic pathways and specific metabolites that contribute to the susceptibility of dairy cows to RP during the dry-off period.

As an emerging omics technique, metabolomics is increasingly being used to explore the etiopathology of various periparturient diseases and identify biomarkers for the early detection, monitoring, and prediction of complex diseases [7,8]. Moreover, metabolomics was employed for intensive investigations of metabolic alterations and biomarker discovery in periparturient diseases of dairy cattle [9,10,11]. In a recent study from our group, carnitine, propionyl carnitine, and lysophosphatidylcholine acyl C14:0 were identified as biomarkers that could predict which cow would develop periparturient diseases, up to −4 weeks before the occurrence of clinical [9,12]. Other research using MS-based metabolomics approaches also revealed that 13 plasma metabolites can serve as potential biomarkers for the diagnosis of ketosis in dairy cows [13]. The same research group reported the involvement of several metabolic pathways in the development and progression of ketosis [14].

Gas chromatography–mass spectroscopy (GC-MS) was widely used in metabolomic studies of periparturient diseases in dairy cows from our group and others [9,10,14,15]. GC-MS is particularly useful for detecting and quantifying organic acids, inorganic acids, amino acids, sugars, and other highly polar compounds. This is because GC-MS methods use chemical derivatization to make these highly polar compounds more separable via gas chromatography. GC-MS is also very good for detecting small volatile compounds (ketones, terpenes, and alkanes) [15,16]. To the best of our knowledge, metabolomics investigations for the prediction of RP have not been conducted previously. In this study, we used a GC-MS metabolomics approach in conjunction with multivariate statistical analysis to study alterations in metabolite profiles during two time points at the dry-off period on the week of diagnosis of RP. The objectives of this investigation were to: (1) determine whether there are alterations in the blood metabolites related to amino acid and carbohydrate metabolism in transition dairy cows, before and during diagnosis of clinical signs of RP; and (2) identify panels of metabolites in the blood of dairy cows that can be used for predicting the risk of cows being affected by RP postpartum.

## 2. Results

**Figure 1:**
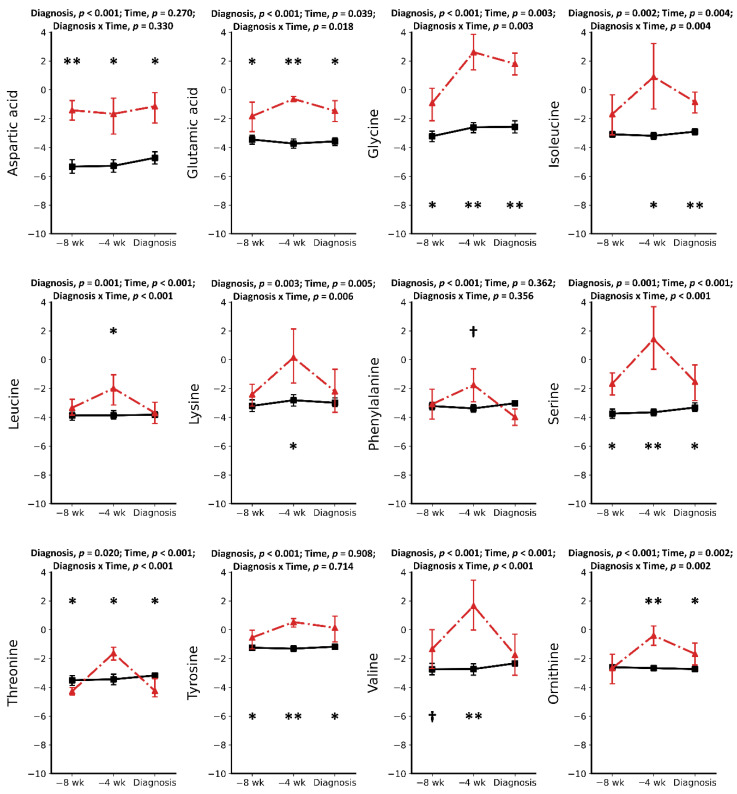
Concentrations of amino acids in the serum of healthy cows (solid line) and pre-retained placenta or retained placenta (dashed line) cows at −8 and −4 weeks prepartum or at the week of retained placenta diagnosis. Asterisks († *p* < 0.1; * *p* < 0.05; ** *p* < 0.001) indicate significant differences between groups at the respective time points. Results are presented in log2.

**Figure 2:**
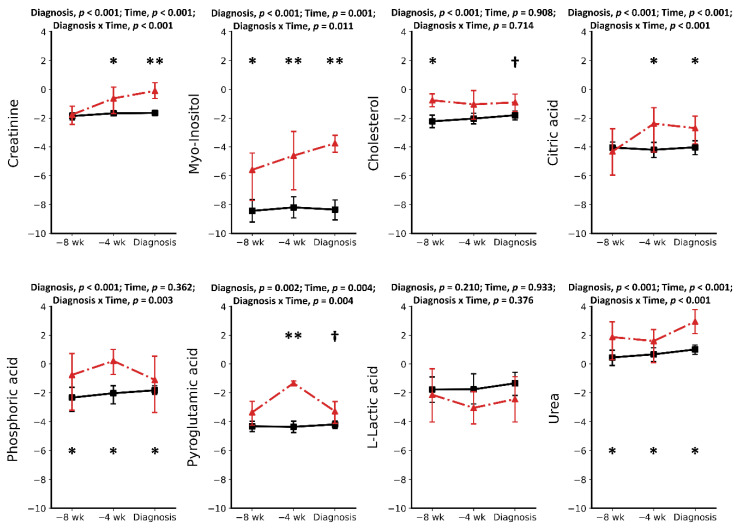
Concentrations of some of the serum metabolites detected in healthy cows (CON) (solid line) and pre-retained placenta or retained placenta (dashed) cows at −8 and −4 weeks prepartum and at the week of retained placenta diagnosis. Asterisks († *p* < 0.1; * *p* < 0.05; ** *p* < 0.001) indicate significant differences between groups at the respective time points. Results are presented in log2.

**Figure 3:**
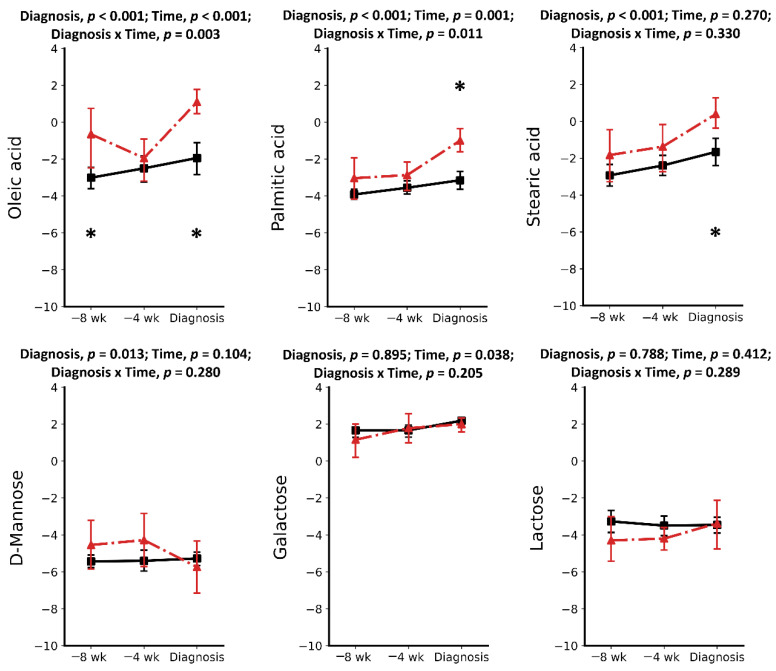
Concentrations of organic acids and carbohydrates in the serum of healthy cows (solid line) and pre-retained placenta or retained placenta (dashed) cows. Asterisk (* *p* < 0.05) indicates significant differences between groups at −8 and −4 weeks prepartum and at the week of retained placenta diagnosis. Results are presented in log2.

Metabolomics analysis using GC-MS was performed on six cases of RP and 16 CON cows. A total of 27 metabolites were identified and quantified in each serum sample using an in-house GC-MS library. Except for cholesterol, urea, and creatinine, the 24 other metabolites can be classified into three groups: amino acids (AAs) (12), organic and inorganic acids (8), and carbohydrates (4). Pre-retained placenta and CON groups were compared at three time points separately, showing that the serum metabolome is altered in pre-RP and RP cows. A combination of univariate and multivariate analyses showed nine metabolites were found to differentiate the two groups at −8 wks, −4 wks, and at the week of disease diagnosis (Figure 1, Figure 2 and Figure 3). The mean ± SD concentration values, *p* values, Log (BF₁₀) along with the fold change and direction of change in RP cases relative to CON cows are provided in Table 1, Table 2 and Table 3.

### 2.1. Metabolic Alterations Prior to Diagnosis of Retained Placenta

Results of this study showed a total of 11 metabolites in the serum of pre-RP cows to be significantly different at −8 weeks prepartum compared to healthy controls. Ten of these metabolites were increased compared with CON cows. Among increased metabolites (Table 1), five were AAs (including Asp, Glu, Gly, Ser, and Tyr) (*p* < 0.05). There were also two organic acids (oleic acid and phosphoric acid), one carbohydrate (myo-inositol), one lipid (cholesterol), and a waste product (urea) that increased in pre-RP cows. The most significantly increased metabolites were Asp, Gly, and myo-inositol with 13.35-, 6.45-, and 6.17-fold change, respectively. In contrast, the only significantly lowered metabolite at −8 weeks prior to parturition was Thr, with a –0.5-fold change (*p* < 0.05).

At –4 wks prepartum, the number of significantly altered metabolites identified and measured was 17. All those metabolites, including Asp, Glu, Gly, Ser, Thr, Tyr, Ile, Leu, Lys, Val, Orn, myo-inositol, phosphoric acid, citric acid, pyroglutamic acid, urea, creatinine, and cholesterol, were found in greater concentrations (*p* < 0.05) in the serum of pre-RP cows. Ser, Ile, and Gly were the most significantly increased metabolites with 151.35-, 88.36-, and 64.09-fold change, respectively (*p* < 0.05).

**Figure 4:**
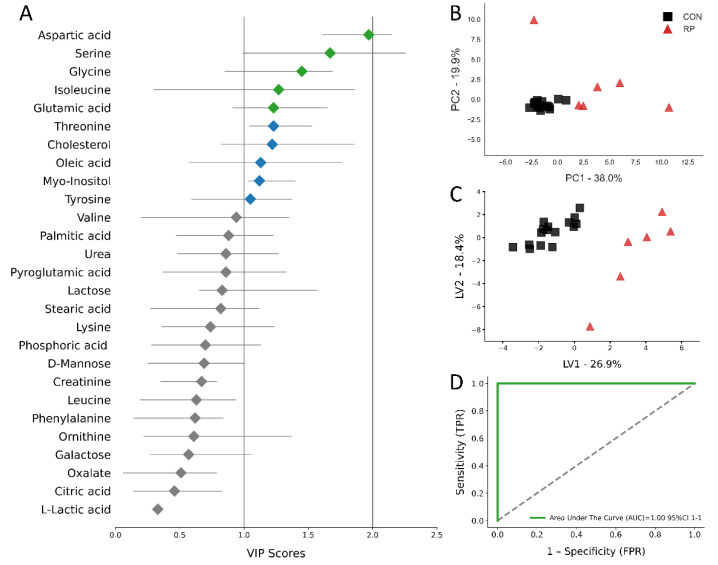
(**A**) Variables ranked by variable importance in projection (VIP), (**B**) PCA, (**C**) PLS-DA (permutation test: *p* < 0.05) of 16 healthy control (CON) cows and 6 cows pre- RP cows at −8 weeks prior to parturition. (**D**) ROC curve for five top-performing metabolites in Vip Scores.

**Figure 5:**
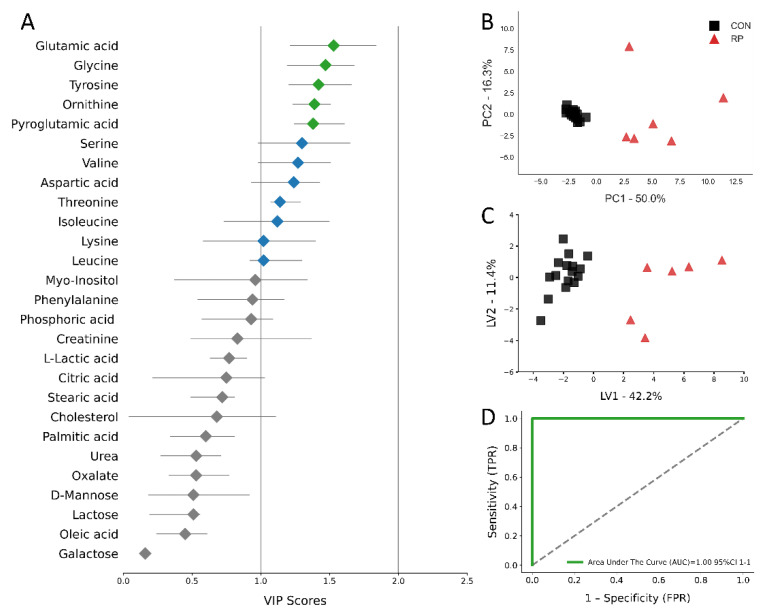
(**A**) Variables ranked by variable importance in projection (VIP), (**B**) PCA, (**C**) PLS-DA (permutation test: *p* < 0.05) of 16 healthy control (CON) cows and 6 pre-RP cows at −4 weeks prior to parturition. (**D**) ROC curve for five top-performing metabolites in Vip Scores.

Multivariate analysis, including PCA and PLS-DA, was used to analyze the GC/MS results. The score plots for the PCA and PLS-DA are shown in Figure 4b,c and Figure 5b,c. All score plots for PCA and PLS-DA at −8 and −4 wks prior to parturition showed a clear separation between CON and pre-RP cows. Of note, the CON group tended to group together, while the pre-RP cows showed a scattered tendency. VIP plots Figure 4a and Figure 5a) present all 27 metabolites that separated both groups of cows. At −8 wks before parturition, the Asp VIP score was (1.97) followed by Ser (1.67), Gly (1.45), Ile (1.27), and Glu (1.23), whereas at −4 weeks prepartum Glu VIP score (1.53) was followed by Gly (1.47), Tyr (1.42), Orn (1.39), and pyroglutamic acid (1.38) (Figure 4a and Figure 5a).

The ROC curves showing the performance of the top five metabolites (by VIP score) are shown in Figure 4d and Figure 5d. The AUC for the two curves was 1.00 (95% CI, 1-1) for both −8 weeks and −4 weeks prepartum. These results demonstrate that biomarker models developed at −8 and −4 weeks could be used to confidently predict which cows are at risk of developing RP postpartum.

Metabolite set enrichment analysis shows the top five metabolic pathways identified as changed at −8 weeks prior to parturition were ammonia recycling, purine metabolism, glutamate metabolism, porphyrin metabolism, and inositol metabolism (Figure 4a). On the other hand, at −4 weeks before parturition, propanoate metabolism, glycine and serine metabolism, arginine and proline metabolism, alanine metabolism, and glutathione metabolism were the top five most important pathways differentiating the pre-RP cows from healthy ones (Figure 4b).

### 2.2. Metabolic Alterations during the Week of Diagnosis of Retained Placenta

A total of 16 out of 27 quantified metabolites in the serum were significantly increased in RP cows, whereas another 2 metabolites were decreased (Table 3) (*p* < 0.05). This included 10 AAs (Asp, Glu, Gly, Ile, Phe, Ser, Thr, Tyr, Orn, and pyroglutamic acid), four organic acids (citric, oleic, palmitic, and stearic acid), one inorganic acid (phosphoric acid), one carbohydrate (myo-inositol), creatinine, and urea. The most significantly increased metabolites were myo-inositol (9.90-fold change) and Gly (8.34-fold change). Threonine and Phe were the two most significantly decreased metabolites (0.63 and 0.59-fold change, respectively) (*p* < 0.05).

**Figure 6:**
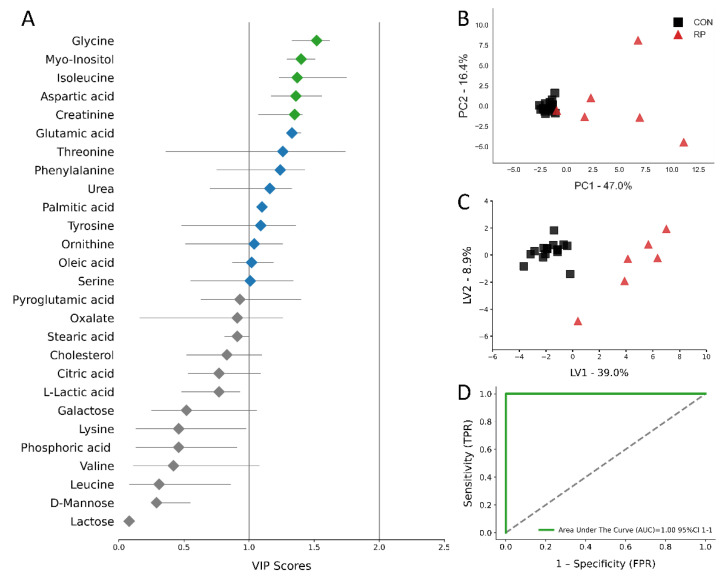
(**A**) Variables ranked by variable importance in projection (VIP), (**B**) PCA, (**C**) PLS-DA (permutation test: *p* < 0.05) of 16 healthy control (CON) cows and 6 RP cows at the week of RP diagnosis. (**D**) ROC curve for five top-performing metabolites in Vip Scores.

At the week of RP diagnosis, the PCA and PLS-DA score plots for the CON and RP groups of cows separate into distinct clusters (Figure 6b,c). The top metabolites with the highest VIP score were Gly (1.52), myo-inositol (1.4), Ile (1.37), Asp (1.36), and creatinine (1.35) (Figure 6a). Those metabolites were used for plotting the ROC curve (Figure 6d), of which AUC was 1.0 (95% CI 1–1). The AUC results indicated that the selected metabolites have excellent predictive value for the diagnosis of RP.

**Figure 7:**
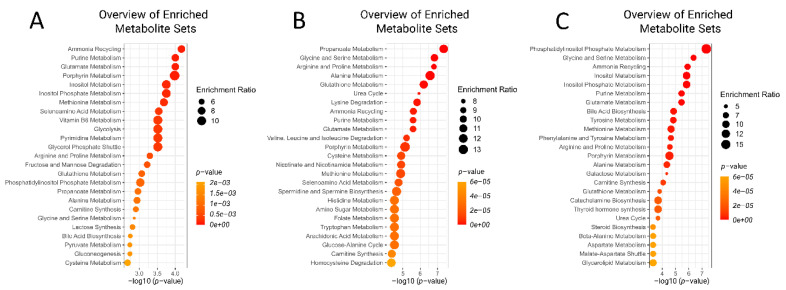
Summary plots for quantitative enrichment analysis at (**A**) −8 weeks and (**B**) −4 weeks prior to parturition and (**C**) week of the diagnosis of retained placenta.

Metabolite set analysis of the GC/MS data indicated that the top five most enriched pathways at the week of retaining placenta were phosphatidylinositol phosphate metabolism, Gly and Ser metabolism, ammonia recycling, inositol metabolism, and inositol phosphate metabolism (Figure 7).

## 3. Discussion

We hypothesized that GC-MS-based metabotyping could be used to identify serum metabolite signatures as well as biochemical pathways altered in pre-RP cows and cows diagnosed with RP. The objective was to identify potential serum biomarkers that can be used to screen cows during the dry-off period to identify those at risk of being affected by RP. Additionally, analyzing metabolic pathways altered in pre-RP cows could give new insights into the pathomechanisms of the disease. Indeed, our results confirmed that pre-RP and RP cows had multiple metabolites and multiple pathways that were altered prior to and during the incidence of RP.

It should be noted that there is not yet a clear understanding of the pathogenic mechanism that contributes to the retaining of fetal membranes. Over the years, several hypotheses have been proposed to explain the etiology of RP. The most important risk factors that have been put forward regarding RP include inflammation, impairment of neutrophil functions, lower phagocytic activity of macrophages in the caruncles, high amounts of endotoxin in the lochia of RP cows, abnormal deliveries, and nutritional deficiencies [17]. Data published by our lab, in a companion article, showed a chronic low-grade inflammatory state in pre-RP and RP cows with higher IL-1, IL-6, TNF, serum amyloid A, and haptoglobin at one or all time points in the study, including −8 and −4 weeks prepartum and during the week of RP diagnosis [5].

The most important finding of the current study was that nine metabolites were found to be consistently and significantly altered at all three time points studied, including Asp, Glu, Gly, Ser, Tyr, Thr, myo-inositol, phosphoric acid, and urea. There were also 4 metabolites (Ile, Phe, citrate, and creatinine) that were altered only at −4 weeks and during RP diagnosis week. In our discussion, we will focus on changes in these consistently altered metabolites.

Two non-essential amino acids, including Asp and Glu, increased in the serum of pre-RP and RP cows versus CON cows. Asp increased 15- and 17-fold at −8 and −4 weeks prepartum and 14-fold at the week of RP diagnosis. On the other hand, Glu increased at a more moderate level (4.30-, 7.69-, and 5.00-fold at −8, −4, and RP week, respectively). It is known that during inflammatory conditions, various nutrients, including amino acids, are released mostly from internal stores to fuel the activation of immune cells [18,19]. Aspartate and Glutamate are important substrates for de novo synthesis of nucleotides that are necessary for the synthesis of both mRNA and proteins [20,21]. Besides their role in protein synthesis, both Asp and Glu have been shown to support immune cells in various ways. For example, Asp serves as a source of carbon skeleton for pyrimidines, which promote the proliferation of activated T cells [22]. Aspartate has also been shown to adjust the expression of pro-inflammatory cytokines by modulating mRNA expression of TLR (toll-like receptors) and NOD genes [23]. Importantly, the greater bioavailability of Asp was demonstrated to promote inflammation by increased production of pro-inflammatory cytokines [24], which is in line with our previously reported findings [5]. On the other hand, Glu acts on iono- and metabotropic receptors expressed on the surface of T and B cells, macrophages, and dendritic cells. Glutamate binding to those receptors triggers a signaling cascade resulting in induction or suppression of leukocyte functions [25]. Additionally, white blood cells use Glu for γ-aminobutyrate (GABA) synthesis [26]. However, the mode of action for Glu is complex and depends on the activity status of the respective immune cells [27]. Concentrations of Glu in the blood are normally relatively low since most of the dietary supply is utilized by enterocytes and the gut microbiome. Therefore, Glu is synthetized either from α-ketoglutarate or other AAs, such as glutamine, arginine, proline, and histidine [28]. Indeed, in a companion paper [6], we found higher Arg and His in RP cows, which might be the result of increased demand for Glu synthesis. Shanshiashvili et al. [29] reported data that showed that Glu might contribute to the formation of a suppressive macrophage genotype. There are two types of macrophages, M1 and M2, and Glu seems to support the M2 phenotype, which is the immunosuppressive phenotype. This suggests that increased Glu might serve to control the scale of the inflammatory response in pre-RP and RP cows.

Threonine, Ser, and Gly were higher in the serum of pre-RP cows at −8 and −4 weeks prepartum (at RP diagnosis week Thr was lower in pre-RP cows). These amino acids share common biochemical pathways; however, only Thr is considered an essential amino acid and cannot be synthetized de novo [30]. Threonine provides benefits to the host in two ways: first, as a major component of mucins, it prevents pathogens from penetrating the enterocytes [31]. Secondly, it is one of the most abundant amino acid in γ-globulin chain [32]. In this regard, Wang et al. [33] showed that increasing dietary Thr enhanced concentrations of antibodies in the blood of pigs. Moreover, as demonstrated by Mawal-Dewan et al. [34], phosphorylation of Thr seems to play an important role in RP recovery via modulation of collagenase expression. Finally, Thr next to Ser regulates protein synthesis since both undergo phosphorylation by mammalian target of rapamycin (mTOR), Ser/Thr protein kinase involved in mRNA translation [35]. Serine and Gly increased in the serum of pre-RP cows by 151- and 64-fold, respectively, at −4 weeks prepartum. The reason for this very high elevation of serum Ser and Gly is not known. Serine and Gly are strongly related compounds because they can be interconverted to each other. They have been proven to play important roles in immunity, especially in macrophages. The latter play significant roles in the detachment of the placenta from caruncles. Rodriguez et al. [36] showed that mice peritoneal macrophages stimulated with LPS rely on Ser to produce Gly that is important for the synthesis of glutathione and the production of IL-1β in vitro. Moreover, Nishiyama et al. [37] demonstrated that activated murine macrophages consume nine amino acids, among which Ser, Glu, Thr. Intriguingly, Ser was consumed at the highest rate, suggesting Ser is a required nutrient for the proliferation and survival of macrophages. Additionally, dietary Gly was shown to improve survival rates and liver functions in endotoxemic rats by regulating both proinflammatory and anti-inflammatory cytokines by inhibiting TNF and stimulating secretion of IL-10 by Kupffer cells in the liver [38]. Taken together, increased serum concentrations of Ser, Gly, and Thr in pre-RP cows is related to supporting the mounting of immune responses, which is in line with our companion article findings [5].

Tyrosine was another amino acid that was higher in pre- and RP-cows at all the time-points studied. Tyrosine derives from the catabolism of amino acid phenylalanine. Tyrosine plays a role in the formation of Glu as well as fumarate and acetoacetate. Interestingly, Tyr concentrations in the serum of CON cows remained almost constant (ranging between 0.42 to 0.45 μmol). On the other hand, Tyr concentrations in pre- and RP-cows doubled from −8 weeks to −4 and during the disease diagnosis week (0.80, 1.50, and 1.63 μmol, respectively). The ratio of Phen to Tyr in human research has been studied thoroughly, and alterations in their ratio have been observed in various inflammatory conditions, including sepsis, cancer, and HIV infection [39]. Indeed, the Phe/Tyr ratio in pre- and RP-cows changed from 0.24 and 0.31 μmol, at −8 and −4 weeks prepartum, to a lower value of 0.04 at RP diagnosis week. This is a 6- to 7.75-fold decrease in the ratio of Phe/Tyr from pre-RP to RP cows. Our data regarding the Phe/Tyr ratio are not in agreement with those in human research, where this ratio has been found elevated during diseases such as sepsis, HIV, and cancer. The discrepancy might be related to the fact that cows in our study were in a chronic low-grade inflammatory state [5], whereas the human subjects were going through far more aggravated inflammatory diseases such as sepsis, HIV, and cancer. Both Phe and Tyr have been shown to influence immune responses. For example, Phe was shown to modulate T cell activity via its oxidative deamination to hydrogen peroxide, which suppresses lymphocytes proliferation [40]. Additionally, Murr et al. [41] demonstrated that serum Phe was associated with activation of the immune response. Phenylalanine controls the antioxidative status of immune cells by regulating the expression of GTP cyclohydrolase I, a key enzyme for NOS (nitric oxide synthase) cofactor synthesis [26]. Phenylalanine is also essential for the synthesis of neurotransmitters and hormones, such as catecholamines. The latter, mainly epinephrine and norepinephrine, are synthetized from Tyr [42]. Norepinephrine has been shown to regulate lymphocyte proliferation, whereas dopamine, which is synthetized from Tyr, has a role in the suppression of the synthesis of proinflammatory cytokines [26,43]. Taken together, the observed alterations in the Phe/Tyr ratios between healthy and RP cows might suggest changes that support an anti-inflammatory response in pre-RP and RP cows.

Our results showed that urea increased in the serum of pre- and RP-cows. Blood urea almost doubled its concentration (1.89-fold higher) from −8 weeks prepartum to disease diagnosis week (from 5.79 to 10.65 μmol) in pre- and RP-cows, whereas concentrations of urea in CON cows ranged slightly between 1.78 to 2.28 μmol in CON cows. Urea is the principal nitrogenous end-product of amino acids and protein metabolism. It is eliminated from the body exclusively from the kidneys through urine. Doubling of blood urea in the pre- and RP-cows suggest a major breakdown of muscle proteins. Besides blood urea, creatinine also was higher in pre- and RP-cows. Creatinine is a waste product produced via catabolism of phosphocreatine in the muscles and is filtered mostly by the kidneys and excreted in the urine [44,45]. Phosphocreatine is used as a source of inorganic phosphorus in the conversion of ADP to ATP in the skeletal muscles [46]. Intriguingly, while the creatinine levels in the blood of CON cows ranged between 0.30 and 0.33 μmol from −8wks prepartum to disease diagnosis, those in pre-RP and RP cows almost tripled (from 0.35 μmol at −8 weeks prepartum to 1.08 μmol at disease diagnosis week) during the same period. Several studies have shown that periparturient cows mobilize muscle protein prior to parturition in kilograms amount. For example, Chibisa et al. [47] showed that periparturient cows mobilized 14 kg of body proteins between d −14 to d 38 postpartum. In a later study, van der Drift et al. [48] found that protein mobilization started prior to parturition. The main question regarding our data is what might have triggered higher protein breakdown in pre- and RP-cows? As already reported in a companion paper, the same pre- and RP-cows were under a chronic low-grade inflammatory state, as indicated by higher IL-1, IL-6, TNF, serum amyloid A, and haptoglobin in those cows. Other research has reported that loss of muscle mass is a common finding in chronic diseases and is associated with elevated concentrations of proinflammatory cytokines, including TNF-*α*, IL-1, IL-6, and IFN-*γ* [49,50,51,52]. It is obvious that the potential cause of higher protein degradation in pre- and RP-cows is the presence of a chronic low-grade inflammatory state and the higher proinflammatory cytokines in the serum of those cows.

Our results showed that pre-RP cows had a greater concentration of myo-inositol (MI) in their serum. Myo-inositol is among nine stereoisomers of inositol and is the most abundant polyol in mammalian cells, synthetized from glucose, mostly in the kidneys [53]. It is involved in various metabolic pathways such as osmoregulation, stabilization of phospholipid membranes, and most importantly, intracellular signaling [54,55]. Intriguingly, MI has been reported as an anti-inflammatory agent, suppressing the production of proinflammatory cytokines and enhancing phagocytic capabilities of macrophages against antibiotic-resistant *E. coli* [56,57]. A recent review by Laganà et al. [58] summarized research indicating MI was able to lower the concentration of IL-6 in a whole variety of inflammatory conditions. Interleukin-6 is a multifunctional cytokine that regulates both humoral and cellular responses, playing an important function in inflammation and tissue damage during infections [59]. It is secreted by Th-1 cells as part of the cytokine storm promoting the recruitment of inflammatory cells at the site of infection [59]. Therefore, elevated concentrations of MI suggest that the host is responding to the presence of a chronic low-grade inflammatory state by releasing blood metabolites with anti-inflammatory properties.

There were four other metabolites that were shared between −4 weeks prepartum and disease diagnosis week, including citrate, creatinine, Ile, and Phe. The first three metabolites were found to be higher in the serum of pre-RP and RP cows, whereas Phe had a bimodal response, increased at −4 weeks prepartum but lowered at RP diagnosis week. There is mounting evidence for the important role of citrate in immune cells. Tannahill et al. [60] and Infantino et al. [61] reported an increase in the concentration of citrate in both mouse (LPS-stimulated) and human (TNF-stimulated) macrophages. Citrate catabolites were linked to secretion of several proinflammatory mediators from LPS- or cytokine-stimulated macrophages, including NO (nitric oxide), ROS (reactive oxygen species), and PGE_2_ (prostaglandin E_2_). Apparently, citrate seems to support the mounting of inflammatory response from the host. Even though creatinine is considered a waste product of creatine-phosphate and biologically an inert catabolite, a study conducted by Riesberg et al. [46] demonstrated that creatinine in vitro was able to suppress TNF mRNA and NF-kB, as well as protein levels in both macrophages and T cell lines, in mouse and human cell lines. This study suggests that creatinine can modulate both innate and adaptive immune responses, which can increase the susceptibility of the host to infections or keep the inflammatory response under control. Regarding Ile, research conducted with weaned piglets showed that oral supplementation with L-Ile enhanced the production of immunoglobulins (IgA and IgG) and some specific antibodies against rotavirus, serum and ileal cytokines (IL-1β, TNF, and IL-10), and β-defensins in the serum, ileum, and mesenteric lymphnodes [62].

Results from this study showed multiple metabolic pathways (25 pathways for each time point studied) that were altered in pre-RP and RP cows. However, there were only 10 metabolic pathways that were shared among the three time points. Out of the 10 metabolic pathways identified as altered, 5 of them involved amino acids metabolism, including Glu metabolism, Gly and Ser metabolism, Arg and Pro metabolism, Meth metabolism, and Ala metabolism. One pathway involved ammonia recycling, another one carnitine synthesis, one included glutathione metabolism, and two others purine and porphyrin metabolism. These data suggest that there is a major involvement of protein mobilization and degradation and release of amino acids in the preceding weeks of parturition in dairy cows at risk of developing RP. As already discussed, some of the metabolites support the proinflammatory responses of the host, and some others play roles as anti-inflammatory agents. The balance between these two opposing metabolic responses determines whether the inflammation will be eliminated or will continue to be present systemically and potentially make cows susceptible to RP. Both immune cell lines were stimulated with bacterial lipopolysaccharide.

Overall, the data from this study demonstrated clearly defined predictive metabolite fingerprints for RP. These are evident with increased serum concentrations of Asp, Glu, Gly, Ser, Thr, Tyr, myo-Inositol, phosphoric acid, and urea at −8 and−4 weeks prior to parturition and at the week of RP diagnosis. Metabolomic analysis during the dry-off period suggests that cows, which are at risk of RP, can be identified during late pregnancy. The metabolites identified in the blood of cows are involved in several important metabolic pathways, including energy supply and immune response. The high predictive accuracy of the top five metabolites at −8 weeks prior to RP (Asp, Ser, Gly, Ile, and Glu) and another five metabolites at −4 weeks prior to parturition (Glu, Gly, Tyr, Orn, and pyroglutamate) suggest that those metabolites may serve as potential monitoring biomarkers for the risk of RP prior to the appearance of any clinical signs. Our results support the idea that metabolomics can provide insights into the etiopathology of RP and might serve to understand better the RP disease process leading to potential therapeutic interventions. However, given the relatively low number of RP cases in our cohort, the results described here should be considered preliminary, and other, larger studies should be conducted to validate the results obtained.

## 4. Materials and Methods

This study was part of a large project designed to expand the understanding of the pathobiology of multiple periparturient diseases in transition dairy cows and to identify potential predictive biomarkers of those diseases. All experimental procedures were approved by the University of Alberta Animal Policy and Welfare Committee for Livestock, and animals were cared for in accordance with the guidelines of the Canadian Council on Animal Care [63].

### 4.1. Animals and Diets

The experimental procedures, management of the animals, diet, and sampling details are described in a companion article [64]. Briefly, 100 pregnant Holstein dairy cows, kept and fed, as described previously [10,65], were used in this nested case-control study. A total of 6 pregnant multiparous (parity: 3.2 ± 0.3, Mean ± SEM) Holstein dairy cows with RP and 20 healthy control cows (CON) that were similar in parity (3.1 ± 0.4; *p* = 0.93) and body condition score (BCS; the mean BCS for both groups was 3.17), were selected. The average age for RP cows was 4.91, and for the CON cows was 4.83. Healthy cows expelled the placenta within 12 h, while in cows diagnosed with retained placenta, placenta remained in the uterus for up to 3 weeks: 4 cows had RP for 1 week and 2 cows had RP for 2 weeks (11 to 17 days). Neither healthy nor RP cows were treated for other diseases. Cows diagnosed with RP were treated with Tetrabol (Vetoquinol N.-A. Inc., Lavatrie, QC, Canada) in the uterine cavity, according to the veterinary protocol.

### 4.2. Blood Sample Collection

In the presented study, blood samples were collected from the coccygeal veins of 26 transition Holstein dairy cows once per week at 0700 before feeding at −8 and −4 weeks prior to parturition and at the day of RP diagnosis. For serum metabolomic analysis, samples from 3 time points: −8 (53–59 d) and −4 (25–31 d) wks before parturition and the disease week from each cow were selected. All blood samples were collected into 10 mL vacutainer tubes (Becton Dickinson, Franklin Lakes, NJ, USA) and allowed to coagulate at room temperature. Immediately after coagulation, the tubes were centrifuged at 2090× *g* at 4 °C for 20 min (Rotanta 460 R centrifuge, Hettich Zentrifugan, Tuttlingen, Germany), and the separated serum was aspirated into a sterile 10 mL plastic test tube (Fisher Scientific, Toronto, ON, Canada). Serum samples were frozen immediately and stored at −80 °C freezer until analyses to avoid loss of bioactivity and contamination and were thawed on ice for approximately 2 h before use.

### 4.3. GC-MS Compound Identification and Quantification

The extraction and derivatization protocol was adapted from a previously reported method to deproteinize and achieve broad metabolite coverage of polar metabolites in serum (PMID: 16351159). Details of sample preparation, injection of derivatized extracts, quality control (QC), raw MS data processing have been previously published [10]. Briefly, 100 μL of serum containing 10 μL of ribitol in ddH_2_O water (0.4 mg/mL), as an internal standard, was extracted with 800 μL of cold HPLC-grade methanol/HPLC ddH_2_O water (8:1 vol/vol) and vortexed for 1 min. The samples were kept at 4 °C for 20 min and then centrifuged at 10,000 rpm for 10 min. After centrifugation, 200 μL of the supernatant was transferred to a glass vial insert (250 μL, Agilent, Santa Clara, CA, USA) in a 1.5 mL glass vial with screw cap (Agilent) and evaporated to dryness using a Speedvac concentrator (Savant Instruments, SDC-100-H, Farmingdale, NY, USA) for 4 h and then using the lyophilizer (LabConco, Kansas City, MO, USA) for 2 h until completely dry.

Derivatized extracts were injected by an Agilent 7683 Series autosampler (Agilent Technologies, Palo Alto, CA, USA). Briefly, A 2 μL aliquot was injected with a 5:1 split ratio onto a 30 m 0.25 mm 0.25 μm DB-5 column (Agilent Technologies). The injector port temperature was held at 250 °C, and the helium carrier gas flow rate was set to 1 mL/min at an initial oven temperature of 50 °C. The oven temperature was increased at 10 °C/min to 310 °C for a final run time of 26 min. Full-scan spectra (50−500 *m/z*; 1.7 scans/s) were acquired after a 6 min solvent delay, with an MS ion source temperature of 200 °C. This was followed by the analysis employing Agilent 6890N GC system coupled with electron impact (EI) ionization mode 5973N mass selective detector (Agilent Technologies, Palo Alto, CA, USA). Raw GC-MS data (“D” file format) were first transformed into CDF format by the ChemStation Data Analysis software (Agilent Technologies, Palo Alto, CA, USA) prior to data pretreatment. Identification and quantification of metabolites were firstly processed and analyzed automatically by a web-based software called GC-AutoFit (http://gcms.wishartlab.com/ (accessed on 20 February 2021), and results were further confirmed manually following the method as previously described [66].

### 4.4. Statistical Analysis

The normality and homogeneity of the distribution of the parameters were tested using the Shapiro–Wilk and Levene’s tests. Our data did not follow a normal distribution. Therefore, univariate analysis of continuous data was performed using the Mann–Whitney U test on log 2 transformed data by R (Version 4.1.0, R Development Core Team, Vienna, Austria, 2008). Assumptions were performed on both log-transformed and non-transformed data. Statistical significance was declared at † *p* < 0.1; * *p* < 0.05; ** *p* < 0.001. In this study, we used log2 transformation for clarity in the graphic representation of data. Data in tables were not transformed to facilitate subsequent meta-analyses that other authors may perform. Additionally, to quantify the strength of evidence for H1 over H0 for univariate analysis, Bayes factor (BF) was calculated using JASP software and presented as Log(BF10). A guide for evaluating log10(Bayes factor) evidence: is [−Inf, 0]—Negative; [0, 0.5]—Weak; [0.5, 1]—Substantial; [1, 1.5]—Strong; [1.5, 2]—Very Strong; [2, +Inf]—Decisive.

According to a previously published protocol, the R-based MetaboAnalyst software and Python-based CIMCB package were used to perform metabolomics data analyses [67]. Metabolites, which, in more than 50% of samples were below the detection limit or were missing in at least 50% of cases, were excluded from the analysis. For those remaining metabolites with missing values, the missing values were replaced by a value of one-half of the minimum positive value in the original data. Before statistical analysis, log-transformation and auto-scaling of the metabolite values were performed as scaling and normalization procedures for all analyzed metabolites.

To perform a standard cross-sectional 2-group study, we compared the healthy cow’s group (control cows, CON) and the cows with the retained placenta (disease group, RP) at each time point −8 weeks, −4 weeks, and disease diagnosis date separately. Time-dependent metabolites changes were analyzed from −8 weeks to disease diagnosis as repeated measures analysis proceeded by a sphericity test. In cases failing the validation of sphericity assumption, the Greenhouse–Geisser correction was used. Metabolite set enrichment analysis was performed via MetaboAnalyst. Principle component analysis (PCA) and partial least squares–discriminant analysis (PLS-DA) was performed using Python packages sklearn, pandas, matplotlib, and seaborn. In the PLS-DA model, a VIP (variable importance in the projection) score was used to rank metabolites based on their importance in discriminating the RP group from the CON group of cows. This was how they were presented on the VIP plots. Validation of each model’s reliability was performed by a 10,000-randomization test. Confidence intervals were estimated using 10,000-step bootstrapping. Receiver-operator characteristics (ROC) were used to illustrate the quality of the 5 biomarkers with higher VIP scores. The area under the ROC curve (AUC) was calculated to estimate accuracy for correctly distinguishing RP cows from CON. A guide for evaluating the utility of a biomarker set based on its AUC was 0.9–1.0 = excellent; 0.8–0.9 = good; 0.7–0.8 = fair; 0.6–0.7 = poor; 0.5–0.6 = fail.

## 5. Conclusions

A targeted GC-MS metabolomics approach was used to identify metabolic changes that precede or are associated with RP in Holstein dairy cows. Our data showed specific metabolite fingerprints that characterized pre-RP and RP cows. Metabolite alterations were identified at both −8 and −4 weeks prepartum as well as during the week of RP diagnosis. A total of 27 metabolites were identified and quantified in each of the serum samples at three time points studied: at −8 and −4 weeks prior to calving as well as the day of RP diagnosis. However, only nine metabolites were consistently altered among the three time points. Those metabolites included five amino acids (Asp, Glu, Gly, Ser, Thr, and Tyr), one sugar (myo-inositol, phosphoric acid, and urea. All nine metabolites were increased in pre-RP and RP cows at all three time points. Four other metabolites altered in pre-RP and RP cows were shared between samples collected at −4 weeks prepartum and at the disease diagnosis week, including citrate, creatinine, and Ile (increased at both time points), and Phe increased at −4 weeks but decreased at disease diagnosis week. The metabolites identified here have the potential to be used as predictive biomarkers of the risk of RP in dairy cows.

## Figures and Tables

**Table 1:** Concentrations of serum metabolites [mean(SD)] in healthy controls (CON) and pre-retained placenta (Pre-RP) cows at −8 weeks pre-partum as determined by GC/MS.

Metabolite. mM	8 Weeks before Parturition					
	Pre-RP	CON	*p*-Value	Log (BF₁₀)	Fold Change	RP/CON
Number of cases	6	16	-	-	-	
Aspartic acid	0.45 (0.12)	0.03 (0.01)	<0.001	7.07	13.35	Up
Cholesterol	0.66 (0.12)	0.28 (0.06)	0.01	2.05	2.35	Up
Citric acid	0.11 (0.04)	0.07 (0.01)	0.35	−0.20	1.50	Up
Creatinine	0.35 (0.07)	0.30 (0.02)	0.30	−0.57	1.19	Up
D-Mannose	0.07 (0.02)	0.03 (0.00)	0.19	2.45	2.74	Up
Galactose	3.03 (0.83)	3.63 (0.38)	0.54	−0.68	0.83	Down
Glutamic acid	0.43 (0.15)	0.1 (0.01)	0.02	3.11	4.25	Up
Glycine	0.80 (0.20)	0.12 (0.02)	0.01	6.74	6.45	Up
Isoleucine	0.68 (0.32)	0.12 (0.01)	0.17	1.73	5.49	Up
Lactose	0.12 (0.06)	0.17 (0.04)	0.18	−0.74	0.70	Down
Leucine	0.12 (0.03)	0.08 (0.02)	0.14	−0.66	1.37	Up
L-Lactic acid	0.80 (0.41)	0.66 (0.15)	0.79	−0.82	1.22	Up
Lysine	0.25 (0.08)	0.14 (0.03)	0.10	0.12	1.84	Up
Myo-Inositol	0.04 (0.01)	0.01 (0.00)	0.01	7.63	6.17	Up
Oleic acid	1.27 (0.44)	0.19 (0.04)	0.02	3.86	6.86	Up
Ornithine	0.25 (0.08)	0.17 (0.01)	0.43	−0.24	1.43	Up
Oxalate	1.71 (0.75)	1.58 (0.41)	0.84	−0.87	1.08	Up
Palmitic acid	0.21 (0.08)	0.07 (0.01)	0.25	1.52	2.85	Up
Phenylalanine	0.19 (0.07)	0.12 (0.01)	0.85	0.08	1.68	Up
Phosphoric acid	1.31 (0.39)	0.35 (0.08)	0.02	3.04	3.75	Up
Pyroglutamic acid	0.13 (0.04)	0.06 (0.01)	0.13	1.03	2.15	Up
Serine	0.41 (0.13)	0.09 (0.01)	0.003	3.68	4.76	Up
Stearic acid	0.63 (0.28)	0.21 (0.05)	0.25	0.85	3.03	Up
Threonine	0.05 (0.01)	0.10 (0.01)	0.01	1.08	0.54	Down
Tyrosine	0.80 (0.14)	0.43 (0.03)	0.02	3.56	1.85	Up
Urea	5.79 (1.46)	1.78 (0.24)	0.01	4.08	3.26	Up
Valine	0.91 (0.46)	0.18 (0.03)	0.09	1.29	5.06	Up

**Table 2:** Concentrations of altered serum metabolites [mean(SD)] in healthy controls (CON) and pre-retained placenta (Pre-RP) cows at −4 weeks pre-partum as determined by GC/MS.

Metabolite. mM	−4 Weeks before Parturition					
	Pre-RP	CON	*p*-Value	Log (BF₁₀)	Fold Change	RP/CON
Number of cases	6	16	-	-	-	
Aspartic acid	0.53 (0.15)	0.03 (0.01)	0.003	6.65	15.97	Up
Cholesterol	0.70 (0.20)	0.29 (0.05)	0.10	1.85	2.39	Up
Citric acid	0.33 (0.07)	0.08 (0.01)	0.01	6.30	4.34	Up
Creatinine	0.87 (0.20)	0.32 (0.02)	0.02	4.65	2.68	Up
D-Mannose	0.10 (0.03)	0.04 (0.01)	0.31	0.45	2.46	Up
Galactose	4.33 (1.02)	3.56 (0.37)	0.35	−0.60	1.22	Up
Glutamic acid	0.65 (0.05)	0.09 (0.01)	<0.001	22.98	7.69	Up
Glycine	12.24 (5.31)	0.19 (0.03)	<0.001	3.39	64.09	Up
Isoleucine	10.43 (5.20)	0.12 (0.01)	0.02	2.51	88.36	Up
Lactose	0.07 (0.02)	0.13 (0.03)	0.25	−0.26	0.52	Down
Leucine	0.36 (0.10)	0.08 (0.02)	0.03	4.23	4.39	Up
L-Lactic acid	0.21 (0.08)	1.03 (0.39)	0.22	−0.34	0.21	Down
Lysine	3.95 (2.02)	0.19 (0.05)	0.01	2.14	20.78	Up
Myo-Inositol	0.09 (0.03)	0.01 (0.00)	0.01	6.82	13.43	Up
Oleic acid	0.39 (0.10)	0.34 (0.11)	0.23	−0.86	1.14	Up
Ornithine	0.96 (0.28)	0.16 (0.01)	<0.001	5.38	6.03	Up
Oxalate	2.79 (0.67)	2.03 (0.43)	0.24	−0.59	1.37	Up
Palmitic acid	0.17 (0.04)	0.11 (0.02)	0.16	−0.08	1.62	Up
Phenylalanine	0.46 (0.13)	0.10 (0.01)	0.05	4.91	4.42	Up
Phosphoric acid	1.58 (0.42)	0.33 (0.05)	0.01	5.32	4.85	Up
Pyroglutamic acid	0.41 (0.03)	0.06 (0.01)	<0.001	19.12	6.53	Up
Serine	12.87 (6.72)	0.09 (0.01)	<0.001	2.27	151.35	Up
Stearic acid	0.64 (0.20)	0.28 (0.06)	0.14	0.87	2.30	Up
Threonine	0.36 (0.07)	0.11 (0.01)	0.001	6.94	3.35	Up
Tyrosine	1.50 (0.16)	0.42 (0.03)	<0.001	14.31	3.55	Up
Urea	4.32 (0.83)	2.05 (0.28)	0.01	2.49	2.11	Up
Valine	10.44 (5.27)	0.18 (0.02)	<0.001	2.40	59.3	Up

**Table 3:** Concentrations of altered serum metabolites [mean(SD)] in healthy controls (CON) and retained placenta (RP) cows at disease diagnosis week as determined by GC/MS.

Metabolite. mM	RP Diagnosis Week					
	RP	CON	*p*-Value	Log (BF₁₀)	Fold Change	RP/CON
Number of cases	6	16	-	-	-	
Aspartic acid	0.73 (0.29)	0.05 (0.01)	0.002	3.64	15.6	Up
Cholesterol	0.63 (0.13)	0.34 (0.05)	0.08	1.24	1.88	Up
Citric acid	0.21 (0.05)	0.08 (0.01)	0.01	3.72	2.78	Up
Creatinine	1.08 (0.25)	0.33 (0.02)	<0.001	5.50	3.26	Up
D-Mannose	0.05 (0.03)	0.03 (0.00)	0.39	−0.42	1.67	Up
Galactose	4.25 (0.58)	4.64 (0.26)	0.85	−0.70	0.91	Down
Glutamic acid	0.45 (0.12)	0.09 (0.01)	0.001	5.46	5.01	Up
Glycine	4.41 (1.10)	0.21 (0.04)	<0.001	8.34	20.72	Up
Isoleucine	0.71 (0.21)	0.14 (0.01)	<0.001	4.62	5.05	Up
Lactose	0.20 (0.10)	0.12 (0.03)	0.58	−0.48	1.66	Up
Leucine	0.10 (0.04)	0.07 (0.01)	0.31	−0.42	1.41	Up
L-Lactic acid	0.49 (0.22)	0.74 (0.18)	0.60	−0.68	0.67	Down
Lysine	0.61 (0.37)	0.15 (0.04)	0.48	0.46	4.04	Up
Myo-Inositol	0.09 (0.02)	0.01 (0.00)	<0.001	9.90	17.60	Up
Oleic acid	2.64 (0.77)	0.56 (0.15)	0.003	3.68	4.70	Up
Ornithine	0.42 (0.15)	0.15 (0.01)	0.02	1.92	2.71	Up
Oxalate	1.99 (0.94)	2.73 (0.46)	0.20	−0.67	0.73	Down
Palmitic acid	0.63 (0.20)	0.16 (0.03)	0.004	2.99	4.02	Up
Phenylalanine	0.07 (0.02)	0.13 (0.01)	0.01	1.94	0.59	Down
Phosphoric acid	1.10 (0.38)	0.33 (0.05)	0.04	2.29	3.36	Up
Pyroglutamic acid	0.13 (0.03)	0.06 (0.01)	0.06	2.14	2.15	Up
Serine	0.59 (0.19)	0.12 (0.02)	0.02	3.42	4.97	Up
Stearic acid	1.83 (0.69)	0.57 (0.14)	0.02	1.40	3.19	Up
Threonine	0.07 (0.03)	0.11 (0.01)	0.02	0.36	0.63	Down
Tyrosine	1.63 (0.61)	0.45 (0.03)	0.01	2.31	3.61	Up
Urea	10.65 (3.47)	2.28 (0.24)	0.006	3.69	4.67	Up
Valine	0.70 (0.33)	0.20 (0.01)	0.31	1.20	3.43	Up

## Data Availability

Data are confidential and not available to the public for intellectual property development reasons.
